# Supplementary material for: Novel NDUFA13 Mutations Associated with OXPHOS Deficiency and Leigh Syndrome: A Second Family Report
Source: Genes (Basel). 2020 Jul 26;11(8):855. doi: 10.3390/genes11080855 (PMC7465247; doi:10.3390/genes11080855)
Supplement: Supplementary file 1 [file genes-11-00855-s001.pdf]

Supplementary Material

Novel *NDUFA13* mutations associated with OXPHOS deficiency and Leigh syndrome: A second family report.

Adrián González-Quintana [1,2], Inés García-Consuegra [1,2], Amaya Belanger-Quintana [3], Pablo Serrano-Lorenzo [1], Alejandro Lucia [4], Alberto Blázquez [1,2], Jorge Docampo [1,2], Cristina Ugalde [1,2], María Moran [1,2], Joaquín Arenas [1,2], Miguel A. Martín [1,2,*].

Supplementary Table 1: Next generation sequencing "OXPHOS panel". Structural subunits and assembly factor genes of OXPHOS system included:

ACAD9	*ATP5J*	*COA1*	*COX18*	*COX7A1*	*LYRM7*	*NDUFA8*	*NDUFB11*	*NDUFS2*	*PET112*	*TMEM126B*
ATP5A1	*ATP5J2*	*COA3*	*COX19*	*COX7A2*	*NDUFA1*	*NDUFA9*	*NDUFB2*	*NDUFS3*	*SCO1*	*TMEM70*
ATP5B	*ATP5L*	*COA4*	*COX20*	*COX7B*	*NDUFA10*	*NDUFAB1*	*NDUFB3*	*NDUFS4*	*SCO2*	*TTC19*
ATP5C1	*ATP5L2*	*COA5*	*COX4I1*	*COX7B2*	*NDUFA11*	*NDUFAF1*	*NDUFB4*	*NDUFS5*	*SDHA*	*UQCC1*
ATP5D	*ATP5O*	*COA6*	*COX4I2*	*COX7C*	*NDUFA12*	*NDUFAF2*	*NDUFB5*	*NDUFS6*	*SDHAF1*	*UQCC2*
ATP5E	*ATP5S*	*COA7*	*COX5A*	*COX8A*	*NDUFA13*	*NDUFAF3*	*NDUFB6*	*NDUFS7*	*SDHAF2*	*UQCR10*
ATP5F1	*ATPAF1*	*COX10*	*COX5B*	*COX8C*	*NDUFA2*	*NDUFAF4*	*NDUFB7*	*NDUFS8*	*SDHB*	*UQCR11*
ATP5G1	*ATPAF2*	*COX11*	*COX6A1*	*CYC1*	*NDUFA3*	*NDUFAF5*	*NDUFB8*	*NDUFV1*	*SDHC*	*UQCRB*
ATP5G2	*ATPIF1*	*COX14*	*COX6A2*	*ECSIT*	*NDUFA4*	*NDUFAF6*	*NDUFB9*	*NDUFV2*	*SDHD*	*UQCRC1*
ATP5G3	*BCS1L*	*COX15*	*COX6B1*	*FASTKD2*	*NDUFA5*	*NDUFAF7*	*NDUFC1*	*NDUFV3*	*SURF1*	*UQCRC2*
ATP5H	*CMC1*	*COX16*	*COX6B2*	*FOXRED1*	*NDUFA6*	*NDUFB1*	*NDUFC2*	*NUBPL*	*TACO1*	*UQCRFS1*
ATP5I	*CMC2*	*COX17*	*COX6C*	*LRPPRC*	*NDUFA7*	*NDUFB10*	*NDUFS1*	*OXA1L*	*TIMMDC1*	*UQCRH*
										UQCRQ

Selection of these genes was carried out by literature and databases search in November 2015.

Supplementary Table 2: Amplification PCR primers for exon 2 and exon 3 of the *NDUFA13* gene.

Exon	Foward Primer	Reverse Primer
2	TGTCACAGTCCAAGCAGCCT	CAACAGCGGAATGCTACAGG
3	GAAAGTAGAAAGCCAACAGTC	AACTCCTAGCTTTTCTCTTGC

Thermocycling program: I) 5 minutes at 95 °C II) 30 cycles: 30 seconds at 94 °C, 30 seconds at 58 °C and 40 seconds at 72°C III) 10 minutes of 72°C
